# Reconsidering the Role of Resting Heart Rate as a Sole Electrocardiographic Predictor of MACE in Women With INOCA

**DOI:** 10.1111/anec.70193

**Published:** 2026-04-24

**Authors:** Ahmet Yılmaz

**Affiliations:** ^1^ Department of Cardiology Karaman Training and Research Hospital Karaman Turkey

Dear Editor,

We read with great interest the recent article by Dungan et al., evaluating electrocardiographic predictors of major adverse cardiovascular events (MACE) in women with ischemia and no obstructive coronary arteries (INOCA) (Dungan et al. 2026). The authors are to be commended for addressing this underrecognized and clinically complex population.

The identification of resting heart rate (RHR) as an independent predictor of MACE is noteworthy; however, several issues merit further consideration. Although statistically significant (HR: 1.018), the effect size is modest, with only an approximately 2 bpm difference between groups, raising concerns regarding its clinical relevance. This suggests that RHR may not represent a causal determinant of adverse outcomes but rather an integrative downstream marker reflecting autonomic imbalance and underlying cardiometabolic burden.

The absence of significant associations for other established ECG parameters, such as QTc interval, QRS duration, and ST‐T abnormalities, is notably discordant with prior literature including earlier WISE analyses (Merz et al. 1999). This discrepancy may be related to methodological factors. Specifically, the use of stepwise regression in a relatively limited event setting raises concerns regarding model stability and potential overfitting. While bootstrapping improves internal validity, it does not fully address issues of generalizability, and relevant ECG predictors may have been excluded due to collinearity or model selection constraints.

Beyond its statistical association, resting heart rate should therefore be interpreted cautiously, as it likely reflects a composite physiological signal integrating sympathetic activation, vascular stiffness, and systemic cardiometabolic stress rather than a direct mechanistic driver of events. In this context, reliance on a single static ECG parameter may oversimplify the complex pathophysiology of INOCA, which is increasingly recognized as a disorder of coronary microvascular dysfunction and systemic hemodynamic dysregulation.

An additional limitation is the lack of heart rate variability (HRV) and ambulatory monitoring data. HRV represents a more sensitive marker of autonomic dysfunction and may provide superior prognostic discrimination compared to static RHR measurements. Similarly, incorporation of dynamic hemodynamic indices—such as blood pressure variability, arterial stiffness, and vascular load parameters—could better capture the multidimensional risk profile of INOCA.

From a clinical standpoint, these findings do not support the use of resting heart rate as a standalone risk stratification tool in INOCA patients. Rather, they highlight the need for integrative, multidimensional models that combine electrocardiographic, autonomic, and hemodynamic parameters.

In conclusion, while this study contributes valuable insights into ECG‐based risk assessment in INOCA, future research should move beyond single‐parameter approaches toward comprehensive risk modeling frameworks that better reflect the complexity of this condition.

## Author Contributions

The author takes full responsibility for this article.

## Funding

The author has nothing to report.

## Ethics Statement

The author has nothing to report.

## Conflicts of Interest

The author declares no conflicts of interest.

## Data Availability

The author has nothing to report.
